# The potential mechanism of celastrol attenuating atherosclerosis by promoting macrophage autophagy via AMPK/ULK1 pathway

**DOI:** 10.3389/fphar.2025.1700663

**Published:** 2025-11-19

**Authors:** Jiao Li, Yanrong Zhao, Yanfang Qi, Yafang Chen, Yue Liu, Linlin Fang, Zhenying Zhou, Liping Wei, Qi Li

**Affiliations:** 1 Department of Cardiology, Tianjin Union Medical Center, The First Affiliated Hospital of Nankai University, Tianjin, China; 2 Department of Internal Medicine, Fugu County Hospital of Traditional Chinese Medicine, Fugu County, Yulin, Shanxi, China; 3 School of Graduate Studies, Tianjin University of Traditional Chinese Medicine, Tianjin, China

**Keywords:** celastrol, atherosclerosis, macrophage, autophagy, AMPK/Ulk1 pathway

## Abstract

**Background:**

This study aimed to elucidate the molecular mechanisms by which celastrol (Cel) alleviates atherosclerosis (AS) through the regulation of macrophage autophagy.

**Methods:**

An AS model was established using ApoE^−/−^ mice fed a high-fat diet. Mice in the treatment group received Cel. Aortic plaque formation, lipid metabolism, inflammatory responses, and autophagy activation were evaluated via histopathological analysis, serological assays, immunofluorescence staining, transmission electron microscopy, and Western blotting. *In vitro*, macrophages were transformed into foam cells using oxidized low-density lipoprotein (ox-LDL) and treated with various concentrations of Cel.

**Results:**

Cel markedly reduced aortic plaque formation, ameliorated dyslipidemia, attenuated inflammatory responses, and enhanced plaque stability in ApoE^−/−^ mice. It significantly promoted macrophage autophagy, as evidenced by increased autophagy-related markers and autophagosome formation. Mechanistically, Cel-induced autophagy was mediated by the AMPK/ULK1 pathway, and this effect was reversed by the AMPK inhibitor Compound C.

**Conclusion:**

Cel exerts anti-atherosclerotic effects by activating macrophage autophagy via the AMPK/ULK1 pathway, thereby improving lipid metabolism, reducing inflammation, and stabilizing plaques. These findings highlight the therapeutic potential of Cel and provide new insights into autophagy-targeted strategies against AS.

## 1 Introduction

Atherosclerosis (AS) is a complex and multifactorial cardiovascular disease that continues to be a major global health burden. It is characterized by the formation of atherosclerotic plaques in the arterial walls, which can lead to plaque rupture, thrombosis, and consequent cardiovascular events. The pathogenesis of AS involves multiple processes, including lipid metabolism disorders, inflammatory responses, and abnormal cellular functions (Libby, 2024).

Autophagy, a conserved cellular self-degradation process, has come to be recognized as a crucial regulator in the development of AS (Miao et al., 2020). It plays a significant role in maintaining cellular homeostasis by removing damaged organelles and misfolded proteins. In the context of AS, proper autophagy in macrophages can prevent the formation of foam cells, a pivotal event in the development of atherosclerotic plaques. However, the dysregulation of autophagy is often associated with the progression of AS. The modulation of cellular autophagy has recently gained prominence as a cutting-edge strategy in AS research. As an essential homeostatic mechanism, autophagy critically regulates the function of multiple cell types within atherosclerotic plaques, including vascular endothelial cells, smooth muscle cells, and macrophages. In endothelial cells, autophagy activation mitigates oxidative and endoplasmic reticulum stress, suppresses the release of inflammatory mediators, and preserves nitric oxide (NO)-mediated vasodilation, thereby maintaining endothelial integrity and delaying AS initiation (Bharath et al., 2017). In vascular smooth muscle cells (VSMCs), enhanced autophagy inhibits the pathological shift from a contractile to a synthetic phenotype, reduces apoptosis, and delays cellular senescence by facilitating the clearance of damaged organelles (Salabei et al., 2013). These effects collectively contribute to the stability of the plaque fibrous cap and help prevent plaque rupture. Macrophages, as central players in AS pathogenesis, exhibit particularly significant autophagic activity. Augmenting autophagy in these cells not only promotes the clearance of lipid droplets via lipophagy—reducing foam cell formation—but also restrains NLRP3 inflammasome activation, leading to decreased secretion of pro-inflammatory cytokines such as IL-1β and IL-18 (Lu et al., 2024). Furthermore, autophagic flux critically influences the stability of ABCA1, a key mediator of cholesterol reverse transport, thereby modulating cholesterol efflux efficiency (Liang et al., 2019). Given these multifaceted roles, targeted regulation of autophagy—especially in macrophages—represents a highly promising therapeutic avenue for AS.

Celastrol (Cel), a natural compound isolated from Tripterygium wilfordii Hook. F., exhibits various biological activities (Xiao et al., 2024). Previous studies have reported its anti-inflammatory, anti-oxidative, and anti-tumor activities (Wang et al., 2025; Zhu et al., 2024). While the broad pharmacological activities of Cel are well-established, its potential role in AS therapy remains underexplored, and its underlying mechanisms are not fully defined. Nevertheless, emerging evidence offers a compelling rationale for its investigation in AS. Notably, Cel has shown efficacy in metabolic disorders such as obesity and diabetes, where it improves systemic glucose and lipid metabolism through activation of key energy sensors including AMPK (Yu et al., 2025). Given the central role of metabolic dysregulation in AS pathogenesis, we hypothesize that Cel’s metabolic benefits may contribute to its potential anti-atherosclerotic effects. AS is also characterized by chronic inflammation. Cel acts as a potent suppressor of pro-inflammatory signaling pathways such as NF-κB, reducing inflammatory responses in multiple disease models. Based on these dual capacities—modulating metabolism and suppressing inflammation, both core features of AS—we propose that Cel may confer anti-atherosclerotic benefits by converging these multimodal effects through a unified downstream mechanism: macrophage autophagy. Understanding how Cel affects AS could potentially lead to the development of novel therapeutic strategies for this prevalent disease. Therefore, this study was designed to systematically investigate the molecular mechanism by which Cell alleviates AS through regulating macrophage autophagy, aiming to provide new insights into the treatment of AS.

## 2 Materials and methods

### 2.1 Animal model

A total of 40 male ApoE^−/−^ mice at 8 weeks of age and weighing 20g∼25 g were purchased from Beijing Huafukang Biotechnology Co., Ltd. [SCXK (JING) 2019-0008]. ApoE^−/−^ mice was genetically engineered to lack the apolipoprotein E gene and were highly prone to AS, were carefully selected. These mice were randomly and evenly allocated into four distinct experimental groups, with each group consisting of ten mice. The study protocol was approved by the Ethics Committee of Tianjin Union Medical Center.

The normal diet group was provided with a standard laboratory chow diet, serving as the control baseline. The high-fat diet group (HFD) was fed a diet containing 21% fat and 0.15% cholesterol for 12 weeks. This is a well-established method for inducing AS in ApoE^−/−^ mice. During this 12-week period, the body weights and general health conditions of the mice were monitored weekly. Body weight was measured using an electronic balance accurate to 0.01 g, and any signs of illness, such as reduced activity, abnormal fur appearance, or changes in eating and drinking behavior, were carefully noted.

After the 12-week diet-induced phase, the success of the AS model was initially evaluated by grossly observing the aorta for visible plaques, followed by histological analysis of aortic sections. Mice were sacrificed under deep anesthesia, and the aorta was carefully dissected. A segment of the aorta was fixed in 4% paraformaldehyde for histological examination.

Subsequently, the atorvastatin group (Ato, 3 mg/kg/d) received daily oral gavage of atorvastatin at a dosage of 3 mg/kg body weight in addition to the high-fat diet. Atorvastatin, a widely used lipid-lowering drug with a definite therapeutic effect on AS, served as the positive drug control. The Cel group (3 mg/kg/d) was given a high-fat diet along with daily oral gavage of Cel at a dose of 3 mg/kg body weight. Mice in the normal and HFD groups were gavaged with sodium carboxymethylcellulose suspension as the vehicle control for 8 consecutive weeks. The Cel used in this study was high-purity powder (purity ≥98%, catalog number: B20707) purchased from Yuanye Bio. For administration, Cel powder was dissolved in a 0.5% sodium carboxymethyl cellulose (CMC-Na) suspension to prepare a 0.5 mg/mL stock solution, which was administered to the mice by oral gavage at a dose of 3 mg/kg/day.

### 2.2 *In vitro* experiment

For the *in-vitro* experiment, RAW264.7 macrophage-derived foam cell models induced by 80 mg/L ox-LDL were utilized. RAW264.7 cells, a murine macrophage cell line, were cultured in Dulbecco’s Modified Eagle’s Medium (DMEM) supplemented with 10% fetal bovine serum (FBS) and 1% penicillin-streptomycin in a humidified incubator at 37 °C with 5% CO_2_. Cells were seeded in 6-well plates at a density of 1 × 10^6^ cells per well. Once the cells reached 70%–80% confluence, they were starved in serum-free DMEM for 12 h to synchronize the cell cycle and then induced with ox-LDL for 24 h. In the *in vitro* experiments, RAW264.7 cells were first treated with 50 μg/mL ox-LDL for 24 h to establish the foam cell model. Subsequently, the ox-LDL-containing medium was removed and replaced with fresh medium containing different concentrations of Cel, followed by further treatment for the indicated durations. The successful induction of foam cells was verified by Oil Red O staining. The *in-vitro* experimental groups included the model group (treated only with ox-LDL), the Cel group (200 nM Cel plus ox-LDL), the AMPK inhibitor Compound C group (5 μM Compound C plus ox-LDL), and the combined intervention group (200 nM Cel and 5 μM Compound C in the presence of ox-LDL).

### 2.3 Oil red O staining​

The dissected aorta was fixed in 4% paraformaldehyde for 24 h. Subsequently, it was cut into transverse sections approximately 2–3 mm thick. These sections were pretreated with 60% isopropanol for 5 min to enhance the penetration of the Oil Red O stain. The sections were then stained with freshly-prepared Oil Red O solution (prepared by dissolving Oil Red O powder in isopropanol and filtering) for 5 min at room temperature. After that, they were counterstained with hematoxylin for 3 min to visualize the nuclei. The area of lipid deposition in the stained sections was quantified using ImageJ software, and the percentage of the lipid-stained area relative to the total aortic cross-sectional area was calculated to assess lipid accumulation.​

### 2.4 HE staining​

Both aortic and liver tissues, upon dissection, were fixed in 4% paraformaldehyde for 48 h. Subsequently, they underwent a series of dehydration steps using graded ethanol solutions (70%, 80%, 90%, 95%, 100% ethanol), were cleared in xylene, and were embedded in paraffin. Paraffin-embedded tissues were sectioned at a thickness of 4–5 μm using a microtome, mounted on glass slides, and stained with hematoxylin-eosin (HE). For aortic sections, the HE-stained slides were examined under a light microscope to analyze the morphological features of atherosclerotic plaques, including the presence of lipid-rich cores, fibrous caps, and inflammatory cell infiltration. In liver tissue sections, HE staining was used to grade hepatic steatosis based on the percentage of hepatocytes containing lipid droplets.​

### 2.5 Masson staining​

Aortic sections, following de-paraffinization and re-hydration, were first stained with Ponceau S solution for 15 min to color the muscle and collagen fibers red. Then, they were rinsed in distilled water and stained with aniline blue solution for 10 min to specifically stain the collagen fibers blue. After being differentiated in 1% acetic acid solution for 3–5 s, the sections were dehydrated, cleared, and mounted. Under a light microscope, the collagen content in atherosclerotic plaques was evaluated by measuring the area of blue-stained collagen fibers relative to the total plaque area. The stability of the plaques was inferred from the distribution and thickness of the collagen rich fibrous caps.​

### 2.6 Immunofluorescence staining​

For aortic frozen sections, the dissected aorta was immediately frozen in optimal cutting temperature (OCT) compound. Sections (6–8 μm thick) cut with a cryostat were mounted on poly-L-lysine-coated slides. After antigen retrieval in a citrate-based buffer (pH 6.0) in a microwave oven for 10–15 min and subsequent cooling, the sections were blocked with 5% bovine serum albumin (BSA) in phosphate buffered saline (PBS) for 1 h at room temperature. They were then incubated with primary antibodies against CD68 (a macrophage marker) and ATG5 (an autophagy marker), diluted in 1% BSA-PBS according to the manufacturer’s instructions, overnight at 4 °C. After three washes with PBS, fluorescent secondary antibodies (Alexa Fluor-conjugated), also diluted in 1% BSA-PBS, were incubated in the dark for 1 h at room temperature. After another three PBS washes, the nuclei were stained with 4′,6-diamidino-2-phenylindole (DAPI) for 5 min. The stained sections were covered with antifade mounting medium and observed under a laser confocal microscope for co-localization analysis of CD68 and ATG5.​

In the *in-vitro* experiment, RAW264.7 cells seeded on glass coverslips placed in 24-well plates were fixed with 4% paraformaldehyde for 15 min, permeabilized with 0.1% Triton X-100 in PBS for 10 min, and blocked with 5% BSA in PBS for 1 h after experimental treatments. They were then incubated with an anti-LC3 primary antibody overnight at 4 °C, followed by incubation with a fluorescent secondary antibody in the dark for 1 h at room temperature after PBS washes. The coverslips were mounted on slides with DAPI-containing mounting medium, and the formation of autophagosomes (appearing as punctate LC3-positive structures) was observed under a fluorescence microscope. The number of autophagosomes per cell was counted in at least 50 cells per experimental group, and the average number was calculated.​

### 2.7 Transmission electron microscopy​

A 1 mm^3^ piece of aortic tissue was carefully dissected and immediately fixed in 2.5% glutaraldehyde in 0.1 M cacodylate buffer (pH 7.4) for 2 h at 4 °C. Subsequently, it was post-fixed with 1% osmium tetroxide in the same buffer for 1 h at 4 °C. The fixed tissue was dehydrated through a series of graded ethanol solutions (30%, 50%, 70%, 80%, 90%, 95%, 100% ethanol), infiltrated with propylene oxide, and embedded in epoxy resin. Ultrathin sections (70–90 nm thick) cut with an ultramicrotome and collected on copper grids were double-stained with uranyl acetate for 15 min and then with lead citrate for 5 min. Autophagosomes, identified by their double-membrane structure enclosing cytoplasmic components, were analyzed for number, morphology, and size using TEM imaging software. The average size and number of autophagosomes per unit area of aortic tissue were calculated.​

### 2.8 Western blot​

Tissues or cells were lysed in RIPA buffer containing protease and phosphatase inhibitors and incubated on ice for 30 min. The lysates were centrifuged at 12,000 rpm for 15 min at 4 °C to pellet the cell debris, and the supernatant containing the total protein was collected. The protein concentration was quantified using the bicinchoninic acid (BCA) method. Protein samples were mixed with loading buffer, boiled for 5 min, and then subjected to sodium dodecyl sulfate‒polyacrylamide gel electrophoresis (SDS-PAGE). The separated proteins were transferred onto PVDF membranes (Bio-Rad, U.S.) using a semi-dry transfer apparatus. The membranes were blocked with 5% non-fat milk in Tris-buffered saline with TBST for 1 h at room temperature, and then incubated with primary antibodies against LC3, p62, AMPK, p-AMPK, ULK1, and p-ULK1 (1:2000), diluted in 5% BSA-TBST according to the manufacturer’s instructions, overnight at 4 °C. After three washes with TBST, they were incubated with horseradish peroxidase (HRP)-labeled secondary antibodies (1:5,000), diluted in 5% non-fat milk-TBST, for 1 h at room temperature. After another three TBST washes, the protein bands were visualized using enhanced chemiluminescence (ECL) reagent, and their intensities were quantified using ImageJ software. Relative protein expression levels were calculated by normalizing the intensities of the target protein bands to that of an internal control, such as GAPDH.

### 2.9 Statistical analysis

SPSS 26.0 was used to analyze the statistical data. All the data are presented as the mean ± standard deviation. Student’s t-test was used to compare two groups. One-way analysis of variance (ANOVA) was used for data from three or more groups if they were normally distributed with uniform variance; otherwise, the Kruskal‒Walli’s test was used. *P* values < 0.05 were considered to indicate statistically significant differences.

## 3 Results

### 3.1 Cel alleviates the pathological damage of AS

**FIGURE 1:**
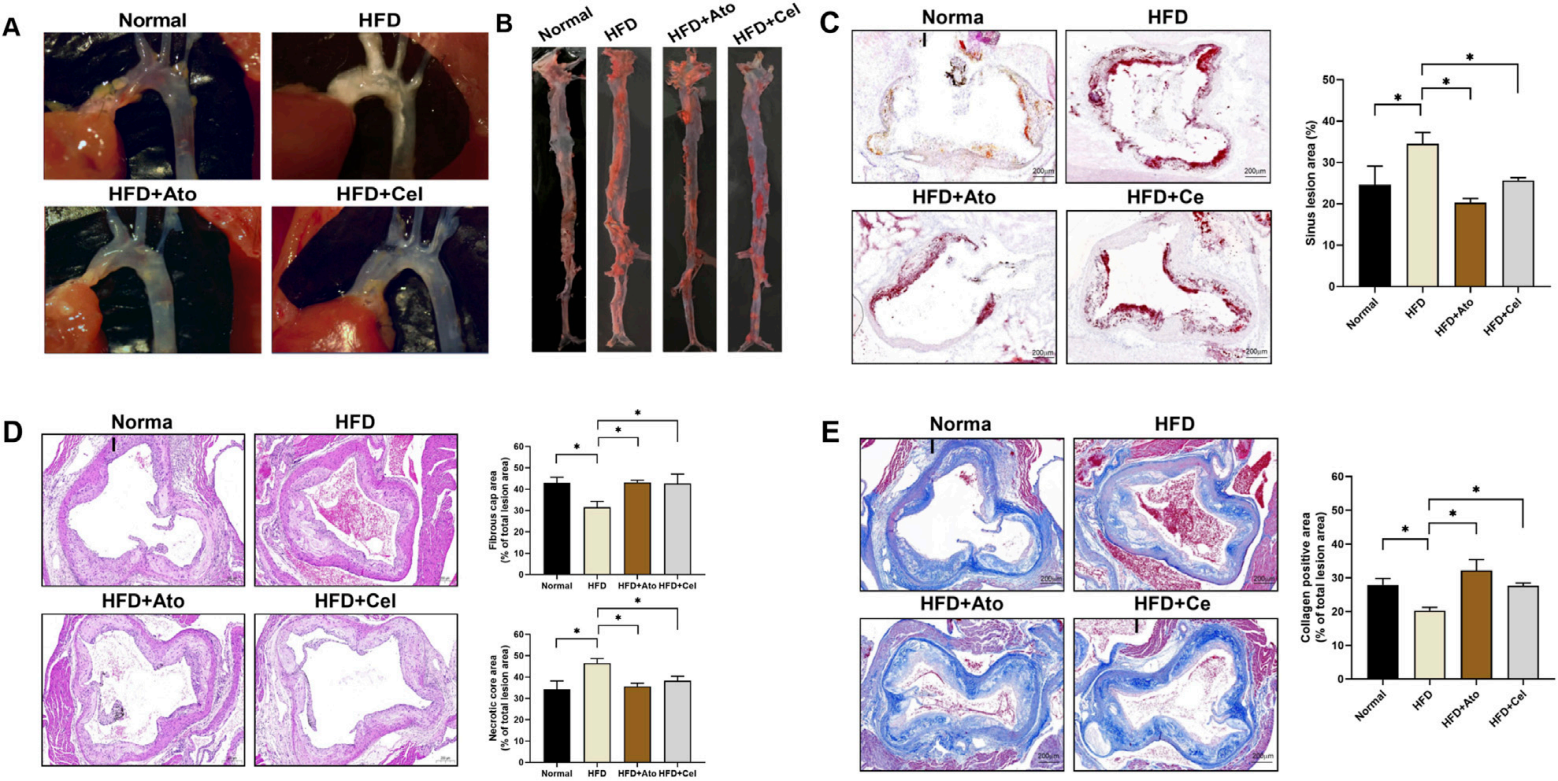
Therapeutic effect of Cel on AS​. **(A)** Stereomicroscopic images showing plaque formation in the aortic arch of mice.​ **(B)** Oil red O-stained entire aorta.​ **(C)** Oil red O staining (50×) of aortic sinus tissue sections and lipid quantification (n = 8).​ **(D)** HE-stained aortic tissue sections (50×), fibrous cap and necrotic core area (n = 8).​ **(E)** Masson-stained aortic tissue sections (50×) and collagen positive area (n = 8).​ *P < 0.05. Data are presented as the mean ± standard deviation. Statistical analysis was performed by paired Student’s t-tests or one-way analysis of variance.

The AS model in ApoE^−/−^ mice was successfully established by a high-fat diet. Pathological analysis showed significant plaque deposition at the aortic arch and its branches in HFD group mice. In contrast, the plaque area in the Cel treatment group was notably reduced (Figure 1A). Oil red O staining of the entire aorta and the aortic root revealed that lipid accumulation in the Cel group was much lower than that in the HFD group (Figures 1B,C). Mouse aortic tissue sections were prepared. HE staining analysis indicated that the area of the necrotic core of plaques in the Cel group was smaller than that in the HFD group (*P* < 0.05), and the thickness of the fibrous cap increased, suggesting improved plaque structural stability (Figure 1D). Masson staining further confirmed that the collagen fiber content in the plaques of the Cel group was significantly higher than that in the HFD group (Figure 1E). These findings suggest Cel can enhance plaque stability by regulating plaque components.

### 3.2 Cel regulates lipid metabolism and inflammatory response

**FIGURE 2:**
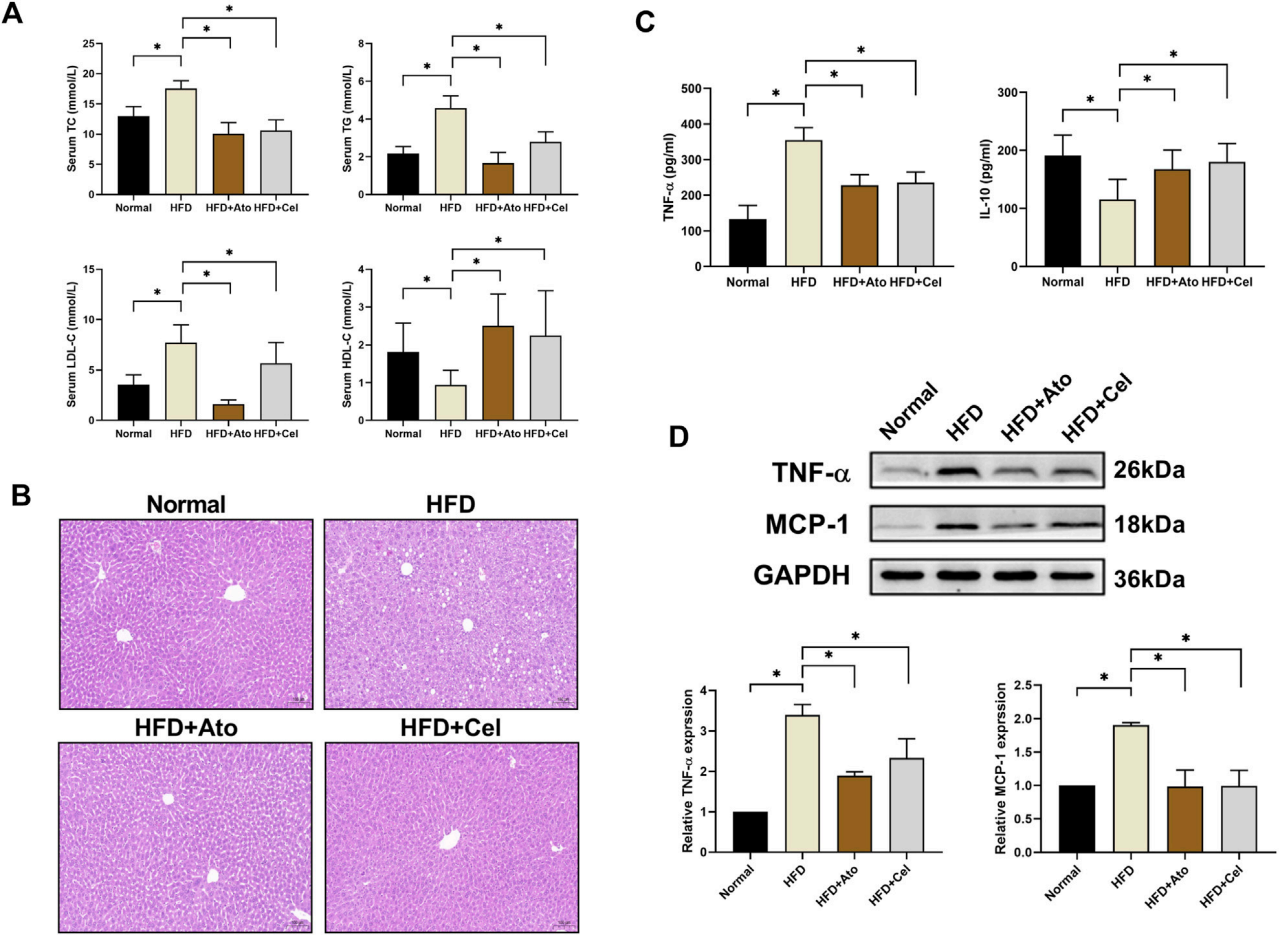
Effects of Cel on lipid metabolism and inflammation​. **(A)** Serum lipid profiles in mice (n = 8). **(B)** HE-stained liver tissues (magnification: ×40).​ **(C)** Serum concentrations of inflammatory factors (TNF-α, IL-10) in mice (n = 8).​ **(D)** Protein expression levels of inflammatory factors (TNF-α, MCP-1) in aortic tissues (n = 8).​ *P < 0.05. Data are presented as the mean ± standard deviation. Statistical analysis was performed by paired Student’s t-tests or one-way analysis of variance.

Serological assays demonstrated that in HFD group mice, the levels of total cholesterol (TC), triglyceride (TG), and low-density lipoprotein cholesterol (LDL-C) were significantly elevated, while the level of high-density lipoprotein cholesterol (HDL-C) was decreased (Figure 2A). After Cel intervention, these indices of lipid metabolism disorders were remarkably improved. Liver pathological examination (Figure 2B) showed that the degree of hepatic steatosis in hepatocytes of the Cel group was milder than that in the HFD group, and the tissue architecture was notably enhanced, suggesting Cel could mitigate steatosis in AS mice. Enzyme-linked immunosorbent assay (ELISA) results indicated that the serum level of the pro-inflammatory factor TNF-α in the Cel group was significantly reduced, while the level of the anti-inflammatory factor IL-10 was increased (*P < 0.05*) (Figure 2C). Western blot analysis further verified that the protein expressions of TNF-α and MCP-1 in aortic tissues of the Cel group were significantly lower than those in the HFD group (Figure 2D), indicating the anti-inflammatory property of Cel.

### 3.3 Cel activates autophagy in macrophages

**FIGURE 3:**
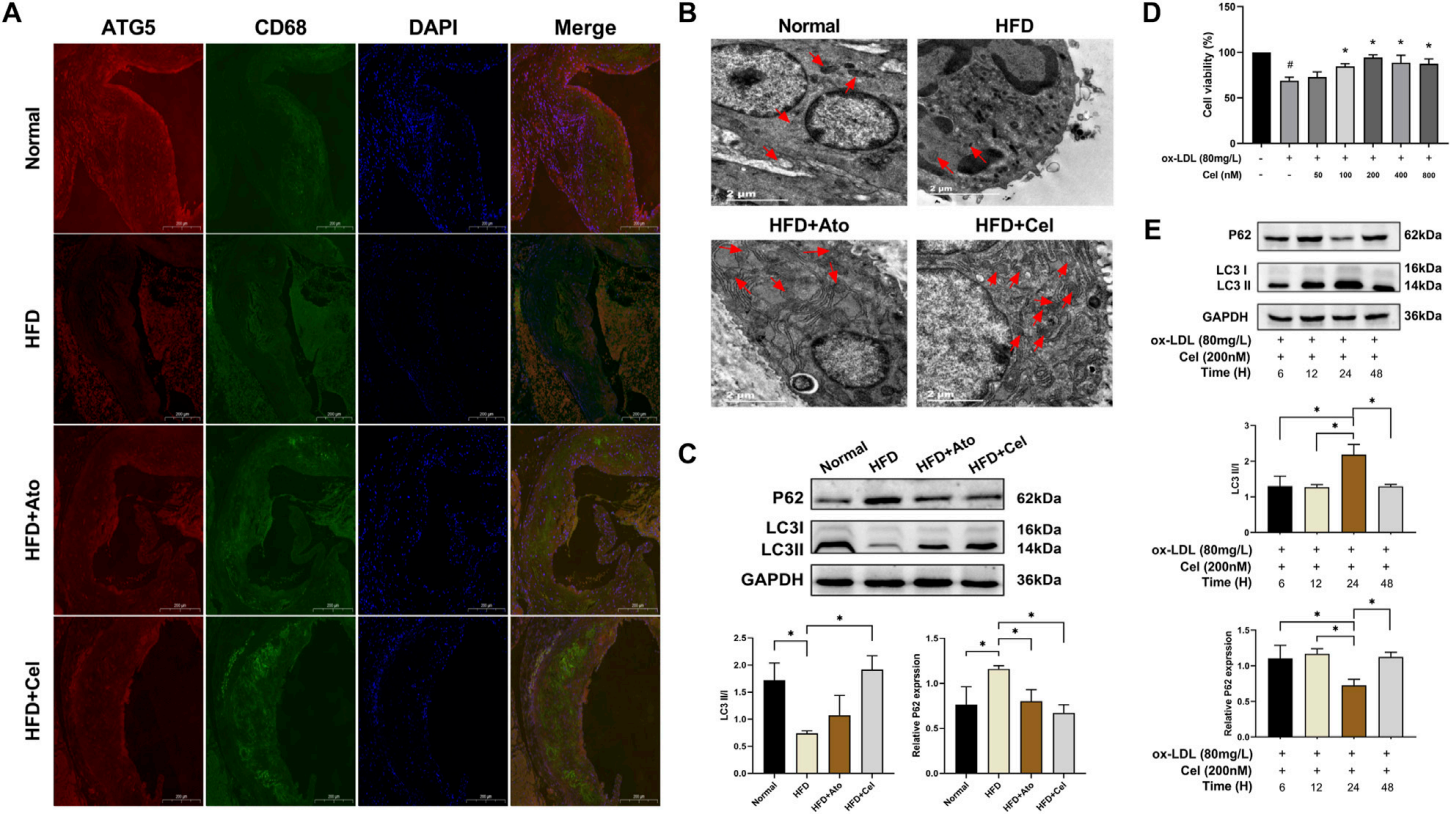
Cel activates autophagy in macrophages​. **(A)** Co-localization of CD68 and ATG5 in aortic tissues (magnification: ×50). **(B)** Visualization of autophagosome formation in aortic tissues by electron microscopy (red arrows denote autophagosomes).​ **(C)** Expression levels of autophagy marker proteins LC3 and p62 in aortic tissues (n = 8).​ **(D)** Cell viability was assessed using the CCK8 assay (n = 3). **(E)** Effects of Cel with different action durations on autophagy-related proteins in foam cells (n = 3). *P < 0.05. Data are presented as the mean ± standard deviation. Statistical analysis was performed by one-way analysis of variance.

Immunofluorescence staining showed that the expression level of ATG5 in CD68^+^ macrophages within aortic plaques of the HFD group was lower than that in the normal group. Conversely, in the Cel group, the expression of ATG5 was restored and had a high degree of co-localization with CD68 (Figure 3A). Transmission electron microscopy analysis revealed that the number of autophagosomes in the Cel group increased significantly (Figure 3B). Autophagosomes typically had a cup-shaped isolation membrane morphology, mainly with a double-membrane structure. Western blot results indicated significant changes in the expressions of autophagy marker proteins LC3 and p62. Compared with the HFD group, the LC3 II/I ratio in the Cel group was significantly elevated, while the protein expression of p62 was decreased (Figure 3C).

In *in-vitro* experiments, macrophages were stimulated with oxidized low-density lipoprotein (ox-LDL) to induce foam cell formation. Subsequently, these foam cells were treated with Cel at different concentrations, and the changes in their viability were monitored. Treatment with ox-LDL resulted in a significant reduction in cell viability compared to the blank control group. However, this ox-LDL-induced decrease in cell viability was reversed by intervention with Cel at various concentrations. Among these, Cel at 200 nM most effectively promoted foam cell growth (Figure 3D). The expression levels of autophagy marker proteins were detected by Western blot. The results showed that Cel could significantly increase the LC3 II/I ratio and reduce the p62 protein content. Notably, the optimal treatment was 200 nM Cel treatment for 24 h, which could remarkably restore the viability of foam cells and activate cellular autophagy (Figure 3E). Therefore, a concentration of 200 nM Cel was selected for subsequent experiments accordingly.

### 3.4 Cel regulates autophagy via the AMPK/ULK1 pathway

**FIGURE 4:**
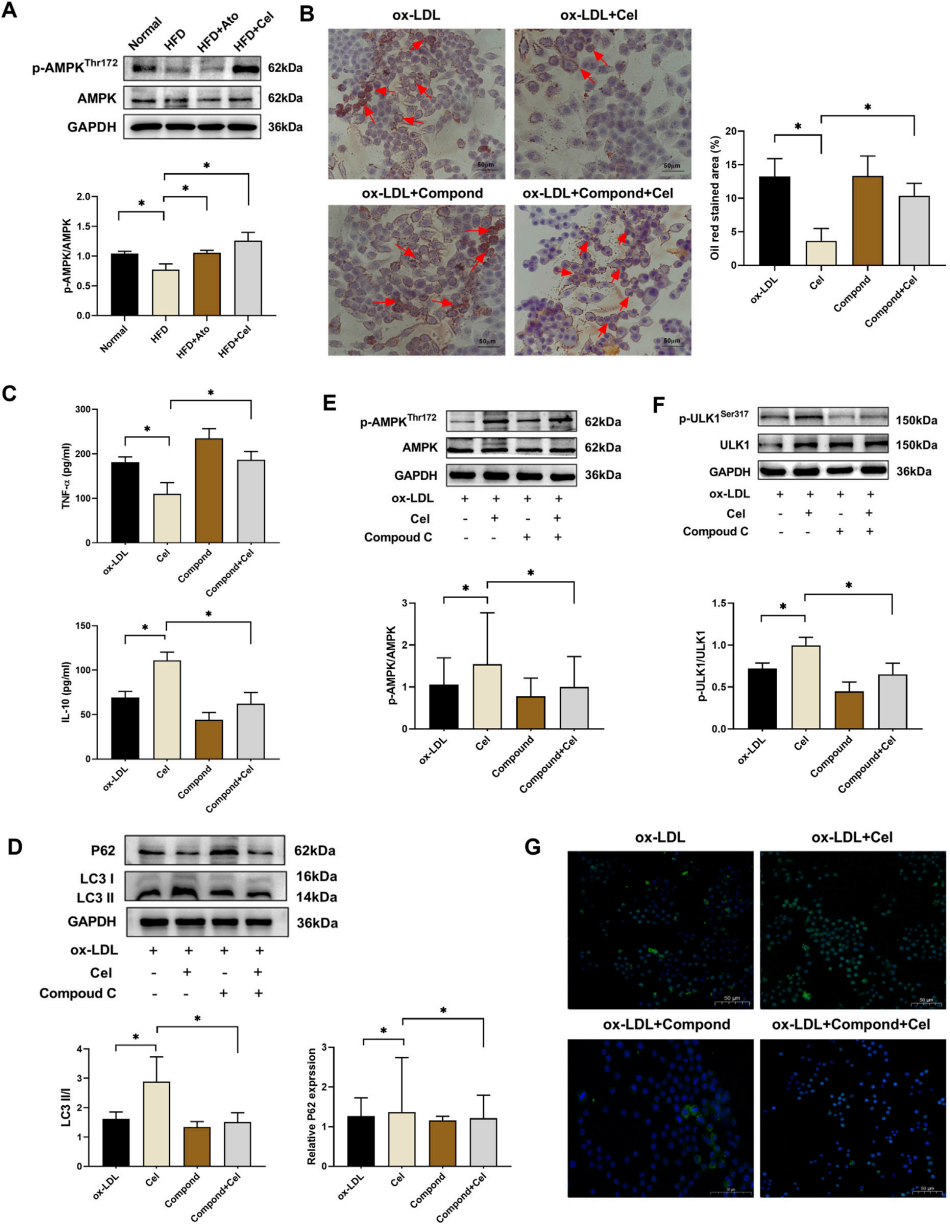
Cel regulates autophagy via the AMPK/ULK1 pathway. **(A)** Expression levels of p-AMPK and AMPK proteins in aortic tissues (n = 8). **(B)** Alterations in lipid accumulation in foam cells (n = 3): Cel markedly decreased red lipid droplets in foam cells. Administration of Compound C reversed Cel’s effect and aggravated lipid-droplet accumulation. **(C)** Levels of inflammatory factors in foam cells (n = 3). **(D)** Alterations in autophagy in foam cells (n = 3). **(E,F)** Expressions of AMPK, p-AMPK, ULK1 and p-ULK1 proteins in foam cells (n = 3). **(G)** Immunofluorescence assessment of the expression of the autophagy-marker protein LC3 in foam cells. *P < 0.05. Data are presented as the mean ± standard deviation. Statistical analysis was performed by one-way analysis of variance.

AMPK, an important kinase regulating energy homeostasis, also acts as a key protein in autophagy regulation. Western blot analysis revealed that the level of phosphorylated AMPK (p-AMPK) in aortic tissues of the Cel group was markedly elevated, while the total AMPK protein level remained unchanged (Figure 4A). When the AMPK inhibitor Compound C was added, it reversed the beneficial effects of Cel on lipid accumulation, inflammatory factor expression, and autophagic activity (Figures 4B–D). Mechanistic studies demonstrated that Cel could significantly enhance the phosphorylation of AMPK and ULK1 in foam cells (*P* < 0.05). However, these effects were abolished after the intervention with Compound C (Figures 4E,F). Immunofluorescence results (Figure 4G) indicated that the fluorescence intensity of LC3 in the Cel group increased substantially. After Compound C treatment, the green fluorescence was notably weakened, and the LC3 expression was significantly reduced. Collectively, these experimental results suggest that Cel exerts its function by activating the AMPK/ULK1 pathway to modulate autophagy.

## 4 Discussion

This study systematically delved into the molecular mechanism whereby Cel promotes macrophage autophagy via the AMPK/ULK1 signaling pathway, thus alleviating AS. The experimental outcomes demonstrated that Cel markedly inhibited the formation of aortic plaques in ApoE^−/−^ mice induced by a high-fat diet. Cel ameliorated lipid metabolism disorders, decreased the levels of inflammatory factors, and enhanced plaque stability by activating autophagy. *In vitro* experiments further validated that Cel regulated macrophage autophagy through the AMPK/ULK1 pathway, reducing lipid-droplet accumulation and the inflammatory response triggered by ox-LDL.

### 4.1 Cel improves the pathological process of AS by activating autophagy

The role of autophagy in AS has garnered extensive attention. Autophagy serves as a crucial cellular self-cleaning mechanism, maintaining metabolic homeostasis by degrading aberrantly accumulated lipids and damaged organelles within cells (Mishra et al., 2025). In the context of AS, when autophagy functions properly, it can effectively clear excess cholesterol esters and malfunctioning mitochondria in macrophages, preventing their transformation into foam cells (Liu et al., 2024). Conversely, autophagic deficiency can hasten the formation of foam cells and plaque inflammation (Mackay et al., 2024).

This study revealed that Cel significantly upregulated the expressions of autophagy markers ATG5 and LC3II/I ratio in the aortic plaques of AS mice while concurrently reducing the p62 protein level. The increase in ATG5 expression indicates enhanced autophagosome nucleation, while the elevated LC3 II/I ratio reflects augmented autophagosome formation (Hosseinzadeh et al., 2025). The reduction in p62, a substrate for autophagic degradation, further validates the acceleration of autophagic flux (Cruz et al., 2025). This suggests that Cel promotes autophagosome formation and the lysosomal degradation process. Transmission electron microscopy further revealed a substantial increase in the number of autophagosomes following Cel intervention, which aligns with literature reports indicating that autophagy activation can curtail lipid deposition and plaque instability (Zhu et al., 2024). Notably, Cel exhibits cell-specific selectivity in autophagy regulation. Immunofluorescence revealed that ATG5 predominantly co-localized with the macrophage marker CD68, indicating that macrophages are the key targets of Cel. Macrophages are abundant in atherosclerotic plaques and play a central role in plaque development. By specifically targeting macrophages, Cel can potentially be developed into a more precise therapeutic approach for AS. This discovery proffers a novel perspective for the treatment of AS by targeting macrophage autophagy.

This study centers on the AMPK/ULK1 signaling axis and its most direct downstream outputs—LC3 conversion and p62 degradations—thereby providing fundamental evidence for Cel-induced autophagy activation in macrophages. Future investigations could expand on these findings by exploring upstream regulatory components, such as the Beclin-1 complex, to achieve a more refined mapping of the signaling network. It should also be noted that autophagic flux was not directly assessed using lysosomal inhibitors such as chloroquine (CQ). Subsequent studies incorporating CQ blockade assays or related methodologies will be valuable to experimentally corroborate the autophagic flux mechanism proposed here.

### 4.2 The AMPK/ULK1 pathway is the core mechanism by which cel activates autophagy

As a pivotal regulatory factor in energy metabolism, AMPK acts as a cellular energy sensor. When the cell experiences energy stress, such as during high-fat diet-induced AS, AMPK is activated. Activated AMPK then initiates the autophagy cascade by phosphorylating ULK1 (Smiles et al., 2024). This study demonstrated that Cel significantly elevated the levels of p-AMPK and p-ULK1 in the aortas of AS mice and in foam cells. The increased phosphorylation levels suggest enhanced activation of the AMPK/ULK1 pathway. Furthermore, the AMPK inhibitor Compound C could reverse its beneficial effects on autophagy and lipid metabolism, corroborating the crucial role of the AMPK/ULK1 pathway. In the absence of Compound C, Cel-induced activation of AMPK leads to increased ULK1 phosphorylation, which in turn triggers a series of events including the recruitment of autophagy-related proteins and the formation of autophagosomes. Previous research has reported that Cel ameliorates mitochondrial function in obesity models via the AMPK/SIRT1 axis (Abu Bakar et al., 2020), and this work for the first time reveals a novel mechanism by which it regulates autophagy through the AMPK/ULK1 pathway in the context of AS. Additionally, the activation of ULK1 may enhance autophagosome assembly by promoting the phosphorylation of Atg13 (Yang et al., 2024). However, the exact sequence of events and potential feedback loops downstream of ULK1 activation remain to be fully elucidated. Future studies could employ techniques such as proteomics and live-cell imaging to precisely map out these downstream molecular events.

A limitation of this study is the absence of an AMPK agonist (e.g., AICAR) as a positive control to further corroborate the role of AMPK from a gain-of-function perspective. Nonetheless, rescue experiments using an AMPK-specific inhibitor confirmed that Cel’s effects depend strictly on AMPK activity, and clear dose-response relationships support the specificity of this mechanism. Collectively, evidence from loss-of-function models and pharmacodynamic analyses provides strong support for the essential role of AMPK. Future studies should incorporate agonist-based controls to establish a conclusive gain- and loss-of-function framework. In addition, genetic knockdown approaches (e.g., siRNA or shRNA) to directly validate the necessity of AMPK or ULK1 represent an important direction for further investigation.

### 4.3 On the dual role of autophagy activation

This study demonstrates that Cel enhances macrophage autophagy via activation of the AMPK/ULK1 signaling pathway, contributing centrally to its atheroprotective effects. However, autophagy acts as a “double-edged sword” in disease contexts (Wang et al., 2021). Moderately activated autophagy serves as a cytoprotective mechanism, clearing misfolded proteins and damaged organelles to maintain cellular homeostasis. In contrast, excessive or dysregulated autophagy can trigger type II programmed cell death—autophagic cell death—compromising tissue integrity and function (Park et al., 2023).

Under our experimental conditions, Cel-induced autophagy exhibited clearly protective features. This conclusion is supported by several lines of evidence: first, the activation of autophagic flux, reflected by an elevated LC3-II/I ratio and p62 degradation, correlated closely with beneficial outcomes including preserved cell viability, attenuated inflammation, and enhanced plaque stability. Second, we detected no significant upregulation of markers associated with excessive autophagy or autophagic cell death, such as prominent cytoplasmic vacuolization. These observations suggest that, in the context of AS, Cel elicits a well-controlled, moderate autophagic response that primarily promotes cellular survival and homeostasis. It remains important to acknowledge that the functional outcome of autophagy is highly context-dependent, influenced by its magnitude, duration, and the specific cellular environment. Further studies are warranted to delineate the dose- and time-dependent effects of Cel on autophagy, and to identify the critical threshold at which protective autophagy shifts toward a detrimental phenotype. Such insights will be essential for evaluating the full therapeutic potential and safety window of Cel as a candidate anti-atherosclerotic agent.

In summary, our findings support a dual role for autophagy in AS, consistent with its “double-edged” nature. This study highlights that moderate, finely-tuned activation of macrophage autophagy through the AMPK/ULK1 pathway represents a key mechanism underlying the plaque-stabilizing effect of Cel. Future work should aim to verify the broader applicability of this concept across diverse disease stages and cellular contexts.

### 4.4 Pleiotropic effects synergistically alleviate AS

Besides activating autophagy, Cel also exerts anti-AS effects by modulating lipid metabolism and suppressing inflammation. The experiments demonstrated that it reduced the serum levels of TC, TG, and LDL-C and increased the level of HDL-C, which is in line with literature findings that it upregulates ABCA1 to facilitate cholesterol efflux. By increasing ABCA1 expression, Cel promotes the transport of cholesterol from macrophages to HDL, thereby reducing intracellular cholesterol accumulation and preventing foam cell formation (Zhang et al., 2021). Simultaneously, Cel markedly inhibited the expressions of pr -inflammatory factors TNF-α and MCP-1 and increased the level of the anti-inflammatory factor IL-10. TNF-α and MCP-1 are known to attract immune cells to the plaque site, exacerbating inflammation and plaque instability (Wu et al., 2024). The increase in IL-10, on the other hand, has anti-inflammatory effects, promoting tissue repair and reducing immune cell infiltration. This coordinated regulation of lipid metabolism and inflammation suggests that Cel intervenes in the core pathological aspects of AS through multiple pathways in a synergistic fashion. Notably, the ameliorative effect of Cel on liver function impairment attests to its safety. In the high-fat diet-induced AS model, liver steatosis is often a comorbidity. Cel’s ability to improve liver function, as evidenced by reduced hepatic steatosis in our study, provides an additional advantage for its potential clinical application.

While this study clarifies how Cel alleviates AS by regulating macrophage autophagy via the AMPK/ULK1 pathway, we recognize a significant limitation: the lack of a wild-type (WT) animal control group to directly assess its potential nonspecific effects in a non-disease context. Nevertheless, thorough re-examination of available data provides indirect support for Cel’s disease model–specific action rather than a broadly nonspecific mechanism.

Our findings reveal a distinct dose–response relationship: ox-LDL–induced suppression of macrophage activity was reversed by Cel in a dose-dependent manner. Importantly, neither insufficient nor excessively high concentrations produced the desired therapeutic outcome—the former being ineffective and the latter leading to effect plateau or potential toxicity. This pattern argues against a nonspecific stimulatory or toxic influence. More critically, pathway rescue experiments using the AMPK-specific inhibitor Compound C markedly reversed Cel-induced autophagy activation, foam cell suppression, and overall anti-atherosclerotic benefits. These results strongly indicate that Cel’s efficacy depends closely on AMPK/ULK1 pathway activation.

We propose that in ApoE^−/−^ mice—characterized by severe dyslipidemia and oxidative stress—macrophage AMPK/ULK1 signaling may exist in a primed or sensitized state, rendering it particularly responsive to Cel. By contrast, under the metabolically stable conditions of wild-type animals, this pathway may exhibit a higher activation threshold, which could explain the lack of comparable phenotypic improvements and further supports the context-dependent specificity of Cel. While the present study confirms AMPK/ULK1 pathway activation in aortic tissues by Cel using Western blot analysis, future work may employ techniques such as immunofluorescence to spatially localize activation of this pathway within specific plaque cell types, such as macrophages and smooth muscle cells.

In conclusion, although direct validation in wild-type models is presently lacking, cumulative pharmacodynamic and mechanistic evidence indicates that Cel’s benefits in AS arise mainly from precise modulation of the AMPK/ULK1 pathway under pathological conditions, rather than from nonspecific effects. Future work will aim to directly test this hypothesis in WT models and evaluate the translational potential of Cel in other metabolic disorders.

Studies have shown that Cel, a natural bioactive compound with relatively low oral bioavailability, can modulate the structure and diversity of gut microbiota (Wang et al., 2024). Hua et al. demonstrated that Cel counteracts obesity by remodeling the gut microbiota under high-fat diet conditions, thereby reducing intestinal lipid absorption (Hua et al., 2021). In a study on hepatocellular carcinoma, Zeng et al. revealed that Cel suppresses tumor growth indirectly via the gut microbiota–bile acid axis, specifically through the *Bacteroides* fragilis–GUDCA–FXR/RXRα–mTOR pathway (Zeng et al., 2023). Similarly, Li et al. reported that in a murine model of ulcerative colitis, Cel alleviates inflammation, restores immune homeostasis, and enhances barrier function in a gut microbiota-dependent manner, mediated through shifts in microbial composition and metabolite profiles (Li et al., 2022).

Building on this evidence, we hypothesize that the systemic anti-inflammatory and lipid-modulating effects of Cel observed in our study may be partly attributable to its ability to remodel gut microbiota. Such modulation could subsequently affect host systemic energy metabolism and immune-inflammatory status via the gut-systemic axis, ultimately acting in concert with its direct activation of the autophagy pathway to ameliorate AS. It should be noted that while the present study focuses on the direct regulatory effect of Cel on the AMPK/ULK1 autophagy pathway and did not examine its influence on gut microbiota, emerging evidence suggests that microbiota modulation may represent an important mechanism underlying the systemic benefits of Cel. This promising avenue warrants further investigation in future studies.

**FIGURE 5:**
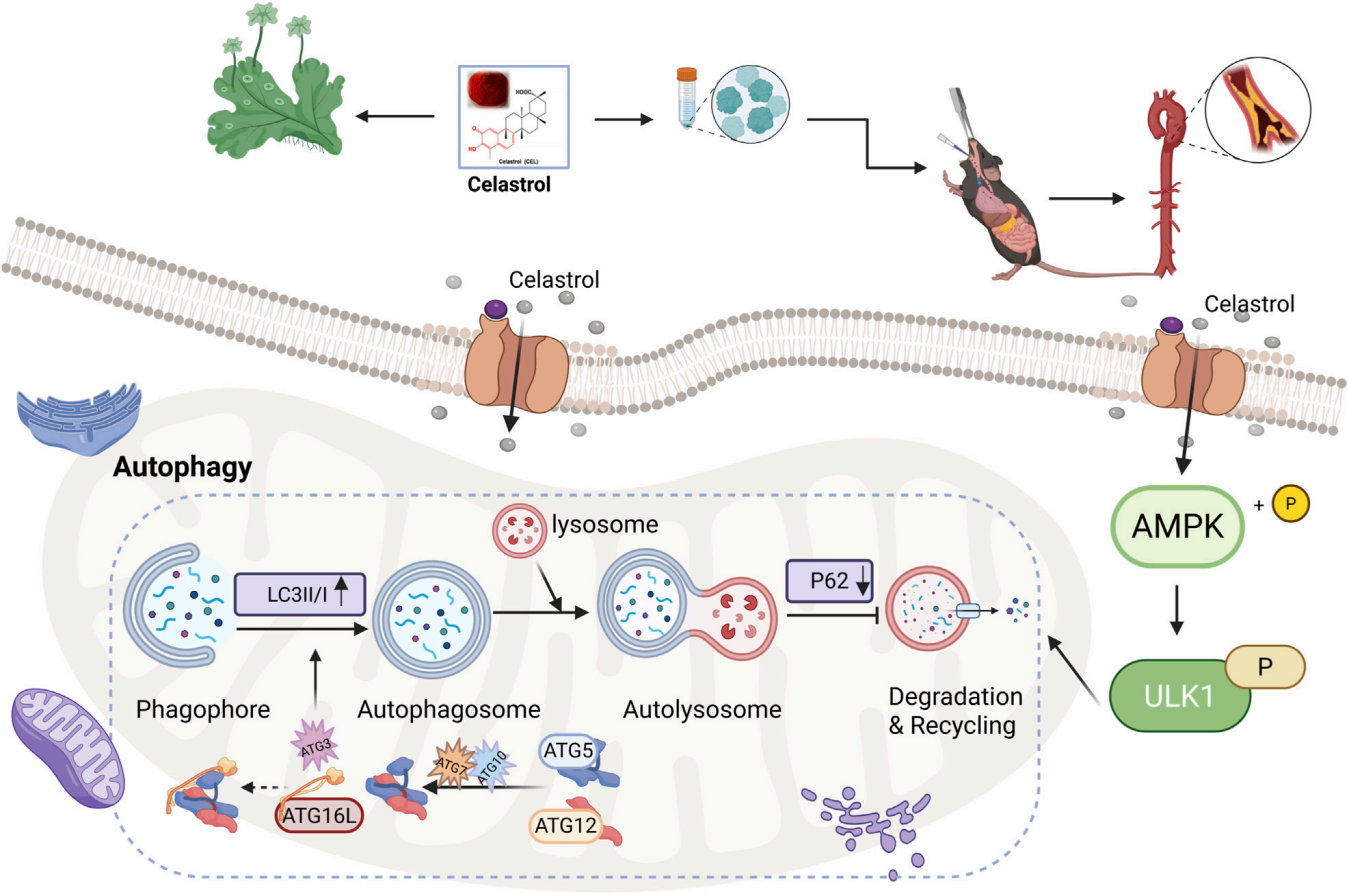
Schematic diagram illustrating the proposed mechanism by which Cel attenuates AS (created with BioRender.com).

Collectively, this study using both *in vivo* and *in vitro* models demonstrates that Cel mitigates AS by activating the AMPK/ULK1 signaling pathway and enhancing autophagic flux in macrophages. The central mechanism we identified is summarized in Figure 5. Specifically, Cel functions as an upstream AMPK activator, leading to ULK1 phosphorylation at Ser555. Activated ULK1 subsequently initiates autophagosome formation by phosphorylating key autophagy-related proteins, a process characterized by conversion of LC3-I to LC3-II and degradation of the autophagy adapter p62. The resulting enhancement of autophagic flux collectively facilitates lipid clearance and attenuates inflammatory responses, thereby reducing plaque burden. Our findings establish AMPK/ULK1-mediated macrophage autophagy as the fundamental mechanism underlying the anti-atherosclerotic effect of Cel.

### 4.5 Limitations and prospects

This study has several limitations that should be acknowledged. First, the validation of the AMPK/ULK1 pathway relied solely on pharmacological inhibitors. Although these tools have provided useful insights, their potential off-target effects necessitate more specific genetic approaches in future work. It will be important to use gene-knockout models—such as mice lacking AMPK or ULK1 in macrophages or other relevant cell types—to more definitively establish the pathway’s role in the mechanism of Cel. A limitation of this study is that the effects of Cel on macrophage viability, autophagy, and the AMPK/ULK1 pathway under normal culture conditions were not examined. Future studies will include this analysis to better define the therapeutic window and safety profile of Cel, and to determine whether its actions depend on a specific pathological stress context.

Second, the dynamic monitoring of autophagic flux did not employ the mRFP–GFP–LC3 dual-fluorescence system, which enables real-time tracking of autophagosome formation and lysosomal fusion. The absence of this methodology may have resulted in the omission of transient autophagic changes. Future studies should incorporate this system to obtain a more complete picture of autophagic activity.

Third, the poor water solubility and bioavailability of Cel remain unresolved. These properties likely limit its effective delivery in both *in vivo* and *in vitro* settings. Subsequent research could explore nano-drug delivery systems to improve solubility and enable targeted delivery to atherosclerotic plaques. Such strategies may enhance therapeutic efficacy while reducing systemic side effects, and should be followed by rigorous pre-clinical pharmacodynamic evaluation.

Finally, the effects of Cel on macrophage activity, autophagy, and the AMPK/ULK1 pathway under normal culture conditions were not assessed. Future studies should examine these baseline effects to better delineate the compound’s therapeutic window and safety profile, and to clarify whether its actions depend on a specific pathological context. In addition, the study lacked *in vivo* dynamic monitoring of vascular plaques using techniques such as ultrasound imaging, relying instead on endpoint pathological assessments. The integration of dynamic imaging in future work will help to clarify the temporal sequence of disease progression and drug effects.

## 5 Conclusion

Cel promotes macrophage autophagy by activating the AMPK/ULK1 pathway, ameliorates lipid metabolism disorders, and suppresses the inflammatory response, thereby stabilizing atherosclerotic plaques. This study provides an experimental basis for Cel as a candidate anti-AS drug and lays a theoretical groundwork for the development of autophagy-regulation therapies.

## Data Availability

The raw data supporting the conclusions of this article will be made available by the authors, without undue reservation.
